# Effector CD4+ T Cell Expression Signatures and Immune-Mediated Disease Associated Genes

**DOI:** 10.1371/journal.pone.0038510

**Published:** 2012-06-08

**Authors:** Wei Zhang, John Ferguson, Sok Meng Ng, Ken Hui, Gerald Goh, Aiping Lin, Enric Esplugues, Richard A. Flavell, Clara Abraham, Hongyu Zhao, Judy H. Cho

**Affiliations:** 1 Department of Medicine, Section of Digestive Diseases, Yale University School of Medicine, New Haven, Connecticut, United States of America; 2 Department of Genetics, Yale University School of Medicine, New Haven, Connecticut, United States of America; 3 W.M. Keck Biotechnology Laboratory, Yale University School of Medicine, New Haven, Connecticut, United States of America; 4 Department of Immunobiology, Yale University School of Medicine, New Haven, Connecticut, United States of America; 5 Department of Epidemiology and Public Health, Yale School of Medicine, New Haven, Connecticut, United States of America; 6 Department of Statistics, Yale University School of Medicine, New Haven, Connecticut, United States of America; 7 Howard Hughes Medical Institute, Yale University School of Medicine, New Haven, Connecticut, United States of America; University of Nebraska Medical center, United States of America

## Abstract

Genome-wide association studies (GWAS) in immune-mediated diseases have identified over 150 associated genomic loci. Many of these loci play a role in T cell responses, and regulation of T cell differentiation plays a critical role in immune-mediated diseases; however, the relationship between implicated disease loci and T cell differentiation is incompletely understood. To further address this relationship, we examined differential gene expression in naïve human CD4+ T cells, as well as in in vitro differentiated Th1, memory Th17-negative and Th17-enriched CD4+ T cells subsets using microarray and RNASeq. We observed a marked enrichment for increased expression in memory CD4+ compared to naïve CD4+ T cells of genes contained among immune–mediated disease loci. Within memory T cells, expression of disease-associated genes was typically increased in Th17-enriched compared to Th17-negative cells. Utilizing RNASeq and promoter methylation studies, we identified a differential regulation pattern for genes solely expressed in Th17 cells (*IL17A* and *CCL20*) compared to genes expressed in both Th17 and Th1 cells (*IL23R* and *IL12RB2*), where high levels of promoter methylation are correlated to near zero RNASeq levels for *IL17A* and *CCL20*. These findings have implications for human Th17 celI plasticity and for the regulation of Th17-Th1 expression signatures. Importantly, utilizing RNASeq we found an abundant isoform of *IL23R* terminating before the transmembrane domain that was enriched in Th17 cells. In addition to molecular resolution, we find that RNASeq provides significantly improved power to define differential gene expression and identify alternative gene variants relative to microarray analysis. The comprehensive integration of differential gene expression between cell subsets with disease-association signals, and functional pathways provides insight into disease pathogenesis.

## Introduction

Genome-wide association studies in immune-mediated diseases have implicated a variety of inflammatory pathways, with a striking overlap of major association signals across disease subtypes [1]. One of the most significant is the interleukin 23 (IL-23) pathway, highlighted by associations within the *IL23R* (interleukin 23 receptor, alpha chain) gene region to inflammatory bowel disease (IBD) [2], psoriasis [3], and ankylosing spondylitis [4]. Interleukin 23 (IL-23) is required for the optimal expansion and maintenance of Th17 lymphocytes, a key pro-inflammatory cell subset [5]. Within IBD, a striking number of the top association signals include genes along the IL-23 signaling pathway, including *STAT3*, *IL12B* (p40, a component of the heterodimeric IL-23 cytokine; also part of IL-12), *JAK2* and *TYK2* [6], [7], [8], although these genes are not specific to IL-23 signaling. In addition, genes enriched in Th17 cells, such as *CCR6*, have also been associated with IBD [8].

Human Th17 cells are characterized by the selective expression of CD161, CCR6 (chemokine C-C motif receptor 6), IL23R and the master transcription factor, RORC [9]. Th17 cells arise from a CD161+CD4+ T cell precursor [10], [11], [12] and CCR6 plays a key role in Th17 cell trafficking to the intestine [13], [14]. Importantly, Th17 cells also expressing IFNγ are abundant in the gut of patients with Crohn’s disease, a subtype of IBD [15]. The traditional view of an initiating role of IFNγ, IL-12 and Th1 cells in immune-mediated human and disease models of autoimmunity has been revised both by human genetic association studies implicating the IL-23 pathway, as well as the requirement for IL-23, and not the IL-12 pathway, in mouse models of intestinal, dermatologic and central nervous system inflammation [16], [17], [18], [19].

Given the preponderance of IL-23 pathway and Th17 enriched genes associated with immune-mediated diseases, we sought to more precisely define the spectrum and molecular basis of differential gene expression in naïve, in vitro differentiated Th1, Th17-negative and Th17-enriched CD4+ memory T cell subsets. We observed an enrichment of immune-mediated disease genes among upregulated genes in CD4+ memory compared to naïve T cells and also among upregulated genes in Th17-enriched CD4+ T cells compared to Th17-negative expanded memory CD4+ T cells. We demonstrate evidence for an abundant, novel isoform in *IL23R* predicted to terminate prior to the transmembrane domain. The differential mechanisms of gene regulation observed between initial Th17-lineage defining genes such as *IL17A, IL17F* and *CCL20*, compared to genes induced in both Th17 and Th1 cells, such as *IL23R* and *IL12RB2,* provide insight into the sequential and versatile roles that Th17 cells play in immune health and disease.

## Results

### The Surface Markers CD161 and CCR6 Distinguish Th17-negative from Th17-enriched CD4+ Memory Cell Subsets

**Figure 1:**
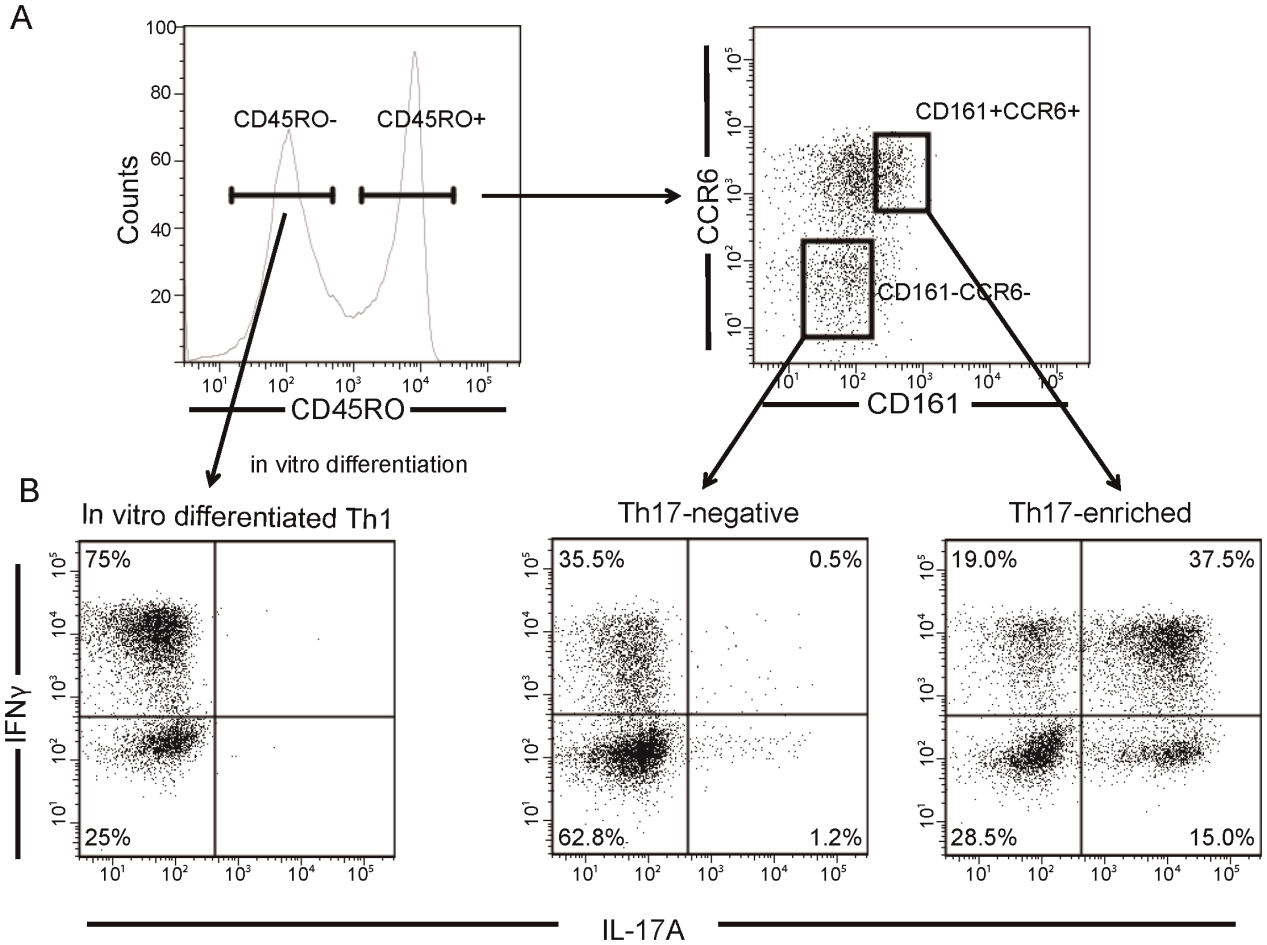
CD4+ T cell subsets. A. CD4+CD25- T cells were sorted by flow cytometry into CD45RO- and CD45RO+ subsets. Memory CD4+CD25-CD45RO+ cells were sorted into CD161+ CCR6+ and CD161-CCR6- subsets for subsequent activation and expansion. B. Th1 cells were generated from naïve CD4+CD62L+CD25-CD45RO- T cells by activating and expanding with anti-CD3, anti-CD28 in the presence of IL-12 and anti-IL4 for seven days. CD161+CCR6+ and CD161-CCR6- memory CD4+ T cells were activated and expanded with anti-CD3, anti-CD28, IL-1β and IL-23 for seven days. The distinct T cell subsets were assessed for differentiation by intracellular cytokine staining for IL-17A and IFNγ expression. Representative flow cytometry plots are shown in the figure.

To explicitly compare Th17-enriched with Th17-negative cells, we sorted freshly isolated human CD4+ memory T cells into CD161+CCR6+ and CD161-CCR6- subsets (Figure 1A), and expanded these cells for seven days with anti-CD3 and anti-CD28 mAb in the presence of IL-23 and IL-1β. Intracellular staining of IL-17 and IFN demonstrates that expanded CD161-CCR6- cells express minimal IL-17 (Figure 1B); in contrast, a significant fraction of expanded CD161+CCR6+ cells produces IL-17 (25–52%, Th17-enriched). Comparable fractions of expanded CD161-CCR6- and CD161+CCR6+ cells express IFNγ; as expected, these fractions are lower than those attained by expansion of naïve CD4+ T cells under Th1-skewing conditions (range, 72–80%) (Figure 1B). Within the expanded memory CD161+CCR6+ subset, 10–15% of cells express IL-17 but not IFNγ, whereas 15–40% of cells express both IL-17 and IFNγ, reflecting heterogeneity of gene expression within Th17-cell subsets (Figure 1B).

### Improved Power to Identify Differential Gene Expression with RNASeq Compared to Expression Microarrays

To accurately evaluate differential gene expression in naïve, in vitro differentiated Th1, Th17-negative and Th17-enriched CD4+ T cell subsets from healthy individuals, we utilized both microarray and transcriptome sequencing (RNASeq) [20] and compared the results. Gene expression was measured by the estimated number of fragments of RNA per kilobase of exon per million fragments mapped (FPKM) for RNASeq and by log_2_ normalized intensity for microarray analysis (Figure S1). The gene expression estimates generated by the two approaches were strongly correlated (r  = 0.83 - 0.85) for all four cell subsets (Figure S1). Of note, the correlation between the two expression estimates was much smaller when restricting to transcripts with lower FPKMs because of the high level of background noise inherent in the microarray platform (r  = 0.38 - 0.45 for transcripts with RNASeq FPKM less than the median).

**Figure 2:**
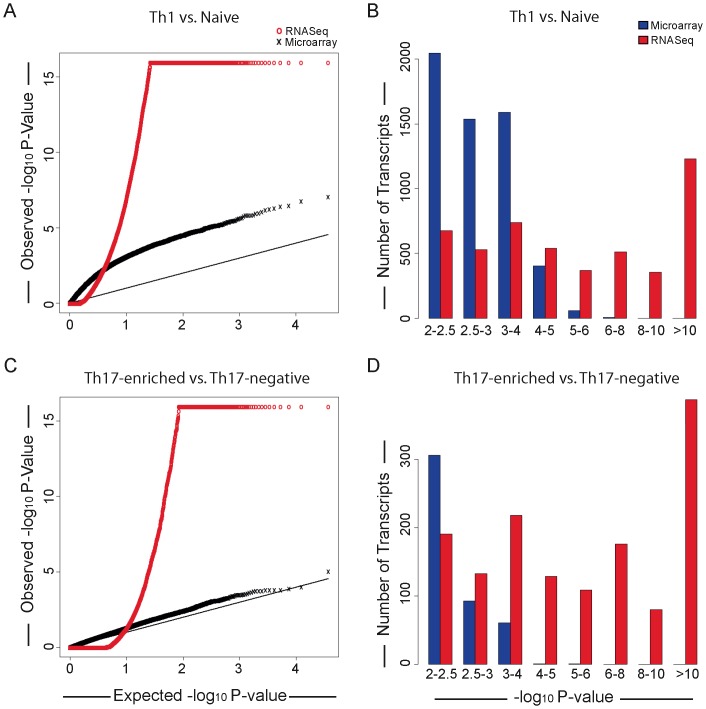
Improved power to identify differential gene expression with RNAseq. A**.** Q-Q plot for observed and expected P-Values for differential gene expression comparing in vitro differentiated Th1 and Naïve CD4+ T cells (N = 4). B. Observed statistical significance for differential gene expression between in vitro differentiated Th1 and Naïve CD4+ T cells (N = 4). C. Q-Q plot for observed and expected P-values for differential gene expression comparing Th17-enriched and Th17-negative CD4+ memory T cells. D. Observed statistical significance for differential gene expression between Th17-enriched and Th17-negative CD4+ T cells.

We next tested for differential gene expression between Th1 vs. naïve and Th17-enriched vs. Th17-negative cells, comparing RNASeq with expression microarrays. The distribution of the P-values obtained from the RNASeq and microarray experiments using Q-Q plots (Figure 2A and 2C) and bar plots (Figure 2B and 2D) demonstrated a marked increase in the number of highly significant differentially expressed genes when expression was measured by RNASeq. Since a comparable number of samples were used for the two approaches (n = 5 for expression microarrays, n = 4 for RNASeq), this indicates that there is markedly greater power to identify differential gene expression using RNASeq compared to expression microarrays. The improved power of RNASeq reflects the numerous sequence reads obtained from analysis of a single sample, compared to the single intensity measurement obtained from microarray expression measurements, thereby allowing the more precise measurement of low transcript abundance compared to microarray-based measurements of gene expression.

### Significant Enrichment for Increased Expression of Genes within Immune-mediated Genetic Loci in CD4+ Memory Compared to Naïve CD4+ T Cells and in Th17-enriched Compared to Th17-negative Expanded Memory CD4+ T Cells

The Immunochip was designed to densely map loci demonstrating genome-wide significant evidence for association to one of nine immune-mediated diseases using 0.1 cM (centimorgan) flanking boundaries from the peak association signal [21]. Loci related to 12 immune-mediated diseases were included on this custom chip. We extracted 1387 genes related to the loci from the Ensembl gene reference set, which we refer to as immune-mediated disease transcript subset. We hypothesized that such genes would demonstrate differential gene expression in CD4+ T cell subsets, since they surround or flank immune-mediated disease loci. Using RNASeq and microarray-based expression in CD4+ T cell subsets, we evaluated differential gene expression for all combinations of CD4+ T cells. We then tested for enrichment of the 1387 Immune-Mediated Disease Transcripts among upregulated genes (defined as those genes having P-values for upregulation amongst the 5% most significant P-values) using Fisher’s exact test for each comparison.

Table 1 reports the enrichment P-values obtained for relative gene expression for each possible pair of cell subsets using both the RNASeq and microarray datasets. First, we observed significant enrichment of immune-mediated disease transcripts among transcripts up-regulated in CD4+ memory T cells compared CD4+ naïve T cells. Specifically, in the RNASeq analysis, significant enrichment of disease-associated transcripts were observed comparing Th17-enriched memory to naïve CD4+ T cells (P-value  = 7.63×10^−04^), with similar, more modest enrichment of Th17-negative memory compared to naïve CD4+ T cells (P-value  = 0.019). Similar, less significant trends are observed in the microarray data; enrichment P-values comparing Th17-enriched memory cells to naïve CD4+ T cells and Th17-negative cells vs. naïve CD4+ T cells 0.025 and 0.091, respectively. Secondly, within memory CD4+ T cells, significant enrichment of immune-mediated disease transcripts was detected in genes upregulated in Th17-enriched compared to Th17-negative CD4+ memory T cells with the RNASeq analysis (P-value  = 2.46×10^−03^). This enrichment was consistently, but less significantly detected in the microarray dataset (P-value  = 0.043). Finally, comparing CD4+ memory T cells and in vitro differentiated Th1 cells, we observed a significant enrichment for upregulated in Th17-negative compared to Th1 (P = 1.13×10^−03^) and Th17-enriched compared to Th1 (P = 7.24×10^−03^) with RNASeq analyses; the P values of the same comparisons in microarray data were rather modest, 0.09 and 0.11 respectively, likely reflecting the lower power of microarray data to identify differential gene expression. Taken together, these data highlight the central role of genes demonstrating enriched expression in CD4+ memory cells generally, and memory Th17 CD4+ T cells specifically, in immune-mediated disease pathogenesis.

**Table 1:** Immune-mediated-associated gene enrichment analysis.

Test	RNASeq	Microarray
Th17-negative>Th17-enriched	0.099	0.52
Th17-enriched>Th17-negative	**2.46E−03**	0.043
Th1>Naïve	0.033	0.67
Naïve>Th1	0.025	0.62
Th17-enriched>Th1	7.24E−03	0.11
Th1>Th17-enriched	0.63	0.95
Th17-enriched>Naïve	**7.63E−04**	0.025
Naïve>Th17-enriched	0.63	0.28
Th17-negative>Naïve	0.019	0.091
Naïve>Th17-negative	0.14	0.95
Th17-negative>Th1	**1.13E−03**	0.09
Th1>Th17-negative	0.24	0.98

Enrichment analysis performed by Fisher’s 2×2 test for the 5% of the most differentially expressed genes between the two compared cell subsets. Bolded are P-values less than 0.004.

As an additional analysis of the enrichment of “Th17” genes (genes upregulated in Th17-enriched memory comparing to Th17-negative memory CD4+ T cells) among autoimmune-disease associated genes, we have identified the known disease associations for each pertinent transcript using the GWAS catalog [22], (Table S1 for RNASeq; Table S2 for microarray data). Genes were selected for this annotation based on satisfying all of the following three criteria: 1) having a P-value less than 0.05 for differential expression between Th17-enriched and Th17-negative memory CD4+ T cells, (2) being upregulated in Th17-enriched cells and (3) appearing in both the microarray and RNASeq datasets. The genes are ordered by P-value for upregulation in Th17-enriched compared to Th-17 memory CD4+ T cells.

**Figure 3:**
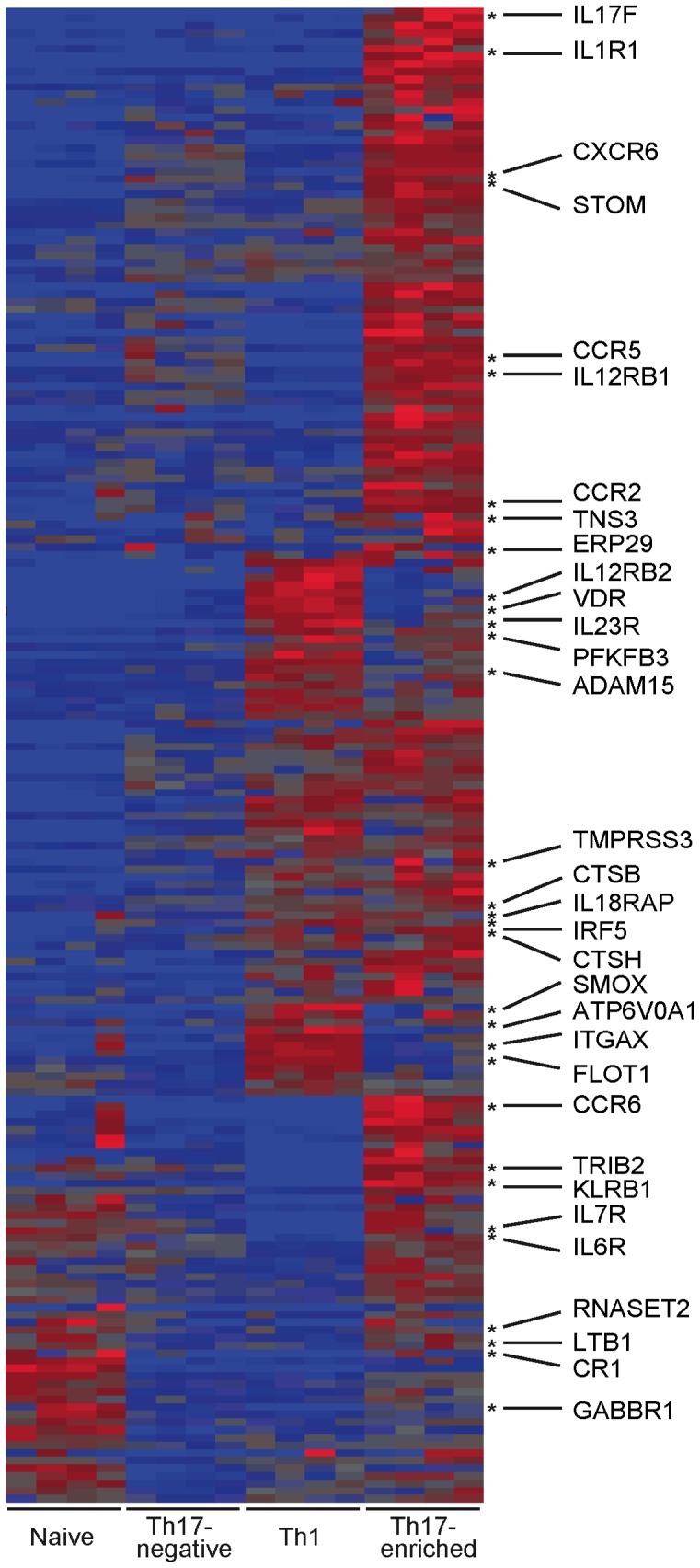
Preponderance of disease-associated transcripts upregulated in Th17 and in vitro differentiated Th1 cells. Hierarchical clustering was performed on 195 transcripts with increased expression in Th17-enriched (red, increased expression relative to other cell types) compared to Th17-negative (blue, decreased expression) and differential expression P-values less than 1×10^−09^. Asterisks (*) designate the transcripts within immune-mediated disease loci. There are total 32 immune-mediated disease associated genes with names marked in the figure.

To further explore the Th17 cell expression signature, we performed cluster analysis focusing on the transcripts demonstrating increased expression in Th17-enriched cells relative to Th17-negative cells by using Partek Genomics Suites (Partek, MO). 195 transcripts with P-values less than 10^−09^ were used for the cluster analysis of the RNASeq dataset (Figure 3), and 147 transcripts with at least a 1.5 fold change and P-values less than 0.05 were used for the clustering analysis of the Microarray dataset (Figure S2). We compared the patterns of expression in Th17-enriched, Th17-negative, CD4+ naïve and in vitro differentiated Th1 cells. As expected, the samples from each of the four cell types clustered together. Because the selection criteria for transcript inclusion was based on increased expression in Th17-enriched compared to Th17-negative cells, increased expression (red-colored predominance) is observed in Th17-enriched compared to Th17-negative cells (blue-colored predominance). Asterisks (*) designate the transcripts within immune-mediated disease regions; there were a total of 32 such transcripts in the whole cluster (Figure 3, indicating that these immune-mediated disease transcripts are highly expressed in Th17-enriched CD4+ T cells. In addition, these immune-mediated disease transcripts are not exclusively expressed in Th17-enriched memory T cells. Nearly half are highly expressed in *both* Th17-enriched memory and in vitro differentiated Th1 cells, for instance, *IL23R, IL12RB2, IL18RAP, and CTSH*. Similarly, in the microarray cluster (Figure S2), there were a total of 25 immune-mediated disease transcripts in the total 147 transcripts. These clustering analyses demonstrate the consistency between RNASeq and microarray gene expression (as can also be seen in Figure S1). Taken together, these data support the conclusion from the enrichment analysis that immune-mediated disease transcripts are enriched among transcripts upregulated in Th17-enriched CD4+ memory T cells.

### Promoter Methylation Patterns Contribute to Differential Gene Expression in CD4+ T Cell Subsets

**Figure 4:**
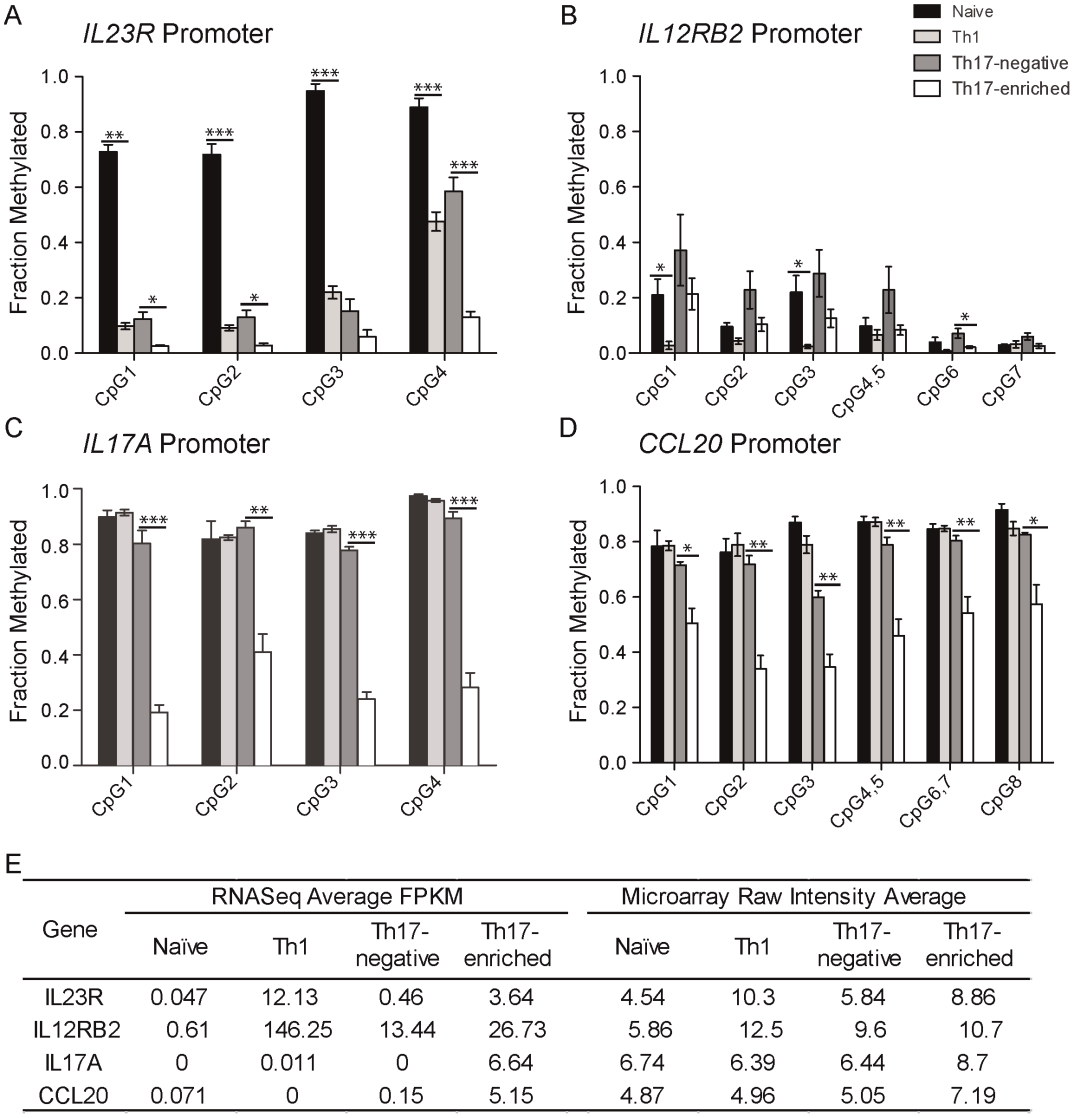
Differential gene expression between CD4+ T cell subsets results from distinct molecular mechanisms. Shown are the fractions of methylation at conserved CpG promoter sites estimated by mass spectrometry (N = 5) for A. *IL23R*, B. *IL12RB2*, C. *IL17A* and D. *CCL20* promoters. Paired t-tests were used to test for differential methylation fractions between naïve vs. Th1 and Th17-negative vs. Th17-enriched were estimated by paired t-test; *P<0.05, **P<0.01, ***P<0.001. E. Average expression estimates from four individulas in both RNASeq and microarray show that Th17-specific transcripts *IL17A* and *CCL20* have nearly zero expression in Th17-negative cells, corresponding to high promoter methylation levels (C–D). Gene expression was measured by FPKM (fragments of RNA per Kilobase of exon per Million fragments mapped) for RNASeq and log2 RMA normalized intensity for microarray.

Gene expression is regulated at multiple levels, including promoter methylation, covalent modification of histones, interactions with non-coding RNAs, and post-translational modifications. We investigated the promoter DNA methylation patterns of illustrative genes in CD4+ T cell subsets, selecting two transcripts demonstrating increased expression in both Th17-enriched and Th1 cells (IL23R and IL12RB2), as well as two transcripts demonstrating increased expression solely in Th17-enriched cells (IL17A, CCL2)), compared with two transcripts demonstrating increased expression (Figure 4, Figure S3). High levels of methylation at conserved CpG sites result in gene silencing [23]; however, additional levels of gene regulation contribute, as gene expression is imperfectly correlated with promoter methylation levels alone [24]. For example, *IL23R* and *IL12RB2* are adjacent on chromosome 1p31; despite similar patterns of gene expression of these two genes across CD4+ T cell subsets (Figure 3), very different patterns of promoter methylation are observed comparing *IL23R* and *IL12RB2* (Figure 4A–B). In the *IL23R* promoter, high levels of CpG methylation and gene silencing are observed in naïve CD4+ T cells (Figure 4A), with significantly lower levels observed in CD4+ memory subsets, including Th17-negative cells that express low levels of *IL23R*. In contrast, *IL12RB2* promoter methylation levels are low in all CD4+ subsets (Figure 4B), even in naïve CD4+ T cells, reflecting a fundamental difference in regulation of gene expression between *IL23R* and *IL12RB2*.

Using RNASeq, differential gene expression between cell subsets associated with the complete absence of mRNA expression can be defined, such as the absence of *IL17A* sequences in Th17-negative subsets; the distinction between the complete absence of gene expression and present, but relatively decreased, gene expression levels, suggests distinct mechanisms of gene regulation. For example, while marked induction of gene expression in Th17-enriched compared to Th17-negative cells is observed for *IL23R*, *IL12RB2*, *IL17A* and *CCL20*, the latter two Th17 signature genes have RNASeq counts in Th17-negative cells near zero (Figure 4E). Consistent with this, high methylation levels (fraction methylated greater than 0.7) are observed at conserved CpG sites within the *IL17A* (Figure 4C), *IL17F* (Figure S3B) and *CCL20* (Figure 4D) promoters in all CD4+ T cell subsets examined except for Th17-enriched cell subsets. Therefore, with *IL17A, IL17F* and *CCL20*, promoter methylation levels account for much of the variable gene expression observed between CD4+ T cell subsets. However, with *IL23R* and *IL12RB2*, promoter methylation levels are less well correlated; for example, modest trends toward decreased CpG methylation (and therefore increased gene expression) in Th17-enriched compared to Th17-negative cells is observed. While CpG promoter methylation levels are comparable between Th1 and Th17-negative cells, *IL23R* gene expression levels are much higher in Th1 cells (Figure 4E).

### Identification of a Major, Novel *IL23R* Truncated Isoform

**Figure 5:**
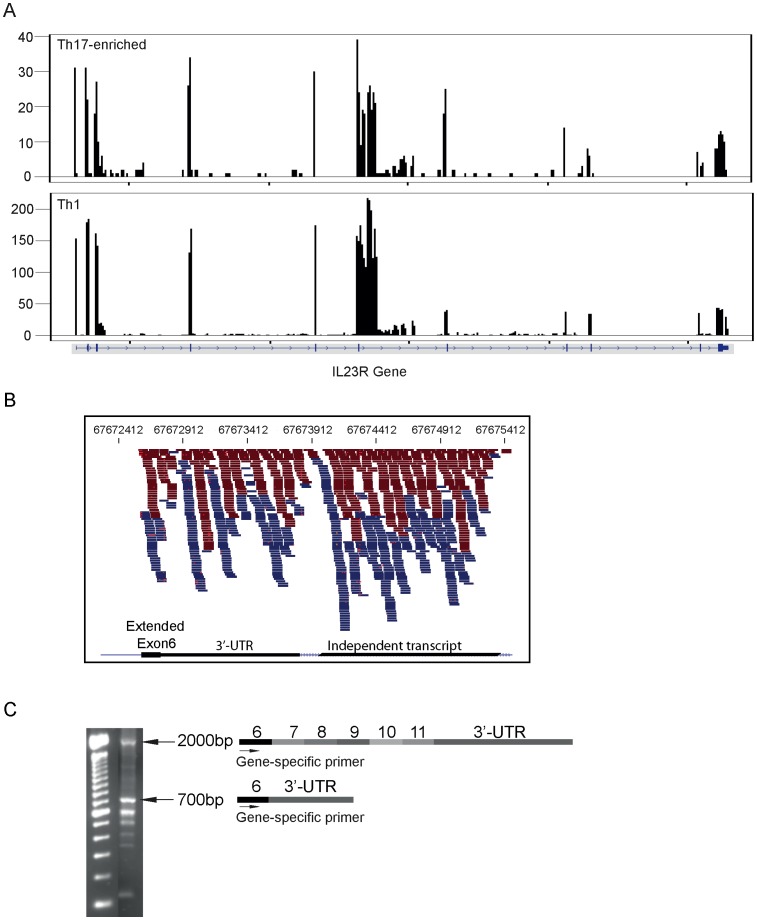
Abundantly expressed, novel *IL23R* isoform. A. Sequence reads were mapped to the *IL23R* gene region in Th17-enriched and in vitro differentiated Th1 cells using Tophat v1.3.3. The intron 6 region was highly covered in both the Th17-enriched and Th1 cell subsets. The scale on the y-axis represents coverage (average number of reads that cover a particular base). B. Zoom in picture of extended coverage on exon 6 and intron 6. Blue and red bars correspond to sense- and anti-sense reads, respectively. The expanded inset demonstrates sequence reads mapping to an extended exon 6 resulting in a stop signal 9 codons downstream with contiguous 3′UTR sequence; in addition an independent transcript maps immediately centromeric to this. C. 3′ RACE (rapid amplification of cDNA ends) was performed, and 2000 and 700 base pair fragments were sequenced, confirming the exon contents designated. The 700 base pair transcript terminating in the intron 6 region would encode for a transcript terminating prior to the transmembrane domain in exon 9.

Inspection of the mapped sequence reads in the *IL23R* gene region on chromosome 1p31 in Th17-negative and positive cells revealed a striking increase of expressed sequences in the intronic region immediately centromeric to exon 6, particularly in Th17-enriched cells and in vitro differentiated Th1 cells (Figure 5A). The y-axis scale represents RNASeq coverage, and indicates that *IL23R* was highly expressed on both Th17-enriched and in vitro differentiated Th1 cells. Also, *IL23R* expression is higher in in vitro differentiated Th1 cells compared to Th17-enriched CD4+ memory T cells. Examination of paired sequence reads (Figure 5B) confirmed numerous examples where the sense sequence originated in the coding portion of exon 6, and its antisense partner mapped to a region centromeric to the exon 6 consensus sequence. Continuation of transcripts into the consensus intron 6 region would result in a stop signal 9 codons downstream of the consensus exon 6 sequence. The transmembrane domain of *IL23R* is contained within exon 9, with the extracellular domain encoded by exons 2 through 8. We performed 3′ RACE priming from the coding region of exon 6 followed by sequence analysis, and confirmed the presence of a major transcript continuing into the region adjacent to the consensus exon 6 region resulting in an extended form of exon 6 (∼700 base pair amplicon, Figure 5C), as well as amplicons containing the full-length transcript (∼2000 base pair fragment, Figure 5C). The presence of PCR amplicons priming between the non-coding region of the extended exon 6 to sense primers in each of exons 2–5 was also confirmed (Figure S4). In the 3′ RACE analysis priming from the coding region of exon 6, the early truncating transcript extends 672 base pairs into the consensus intron 6 region (67,672,739 to 67,673,410 base pairs). In four donors, using Cufflinks applied to a custom annotation file for the *IL23R* region, we observed a high fraction of this *IL23R* transcript with an extended form of exon 6, (32.78% ±4.70% for Th17-enriched and 42.80% ±0.28% for in vitro differentiated Th1 cells). The sequences mapping immediately centromeric to this are largely self-contained, with sequence pairs mapping between 67,673,962 to 67,675,317 base pairs, forming an independent transcript (Figure 5B, zoom-in picture of intron 6). This independent RNA is expressed in both Th17-enriched and in vitro differentiated Th1 cells (Figure 5A), highlighting both the abundance and regulated nature of such novel transcripts.

## Discussion

### Enrichment of CD4+ T Cell Gene Signatures in Immune-mediated Disease Loci

The overlap of associated genetic loci identified by GWAS across immune-mediated diseases suggests a shared pathogenesis. Commonly, the precise risk alleles are also the same, suggesting that the overlap results not merely from the identification of key immune-mediated genes with functional polymorphisms, but that the directionality of effects is also shared. In this study, we observe that genes contained within immune-mediated disease genetic loci demonstrate a striking enrichment of increased gene expression in Th17-enriched, Th17-negative CD4+ memory compared to naïve CD4+ cells (Table 1). Within memory CD4+ T cells, we observe increased gene expression in Th17-enriched compared to Th17-negative cells. Importantly, enrichment of increased gene expression in the converse direction is not observed; for example, genes demonstrating increased gene expression in naïve CD4+ T cells compared to memory T cells are not enriched among disease-associated genes. These findings provide a basis for an increasingly refined and systematic, directional molecular mapping between disease associations and altered function of CD4+ T cell subsets.

It might be anticipated that a Th17 or Th1 enrichment signature would be obscured by the broad inclusion of all transcripts within association signals, as clearly not all genes in associated regions contribute to disease pathogenesis. The presence of a clear Th17 and Th1 expression signature highlights the precise and significant enrichment provided by the composite information gleaned from GWAS across immune-mediated diseases. Furthermore, the commonly observed concordance of expression for genes closely linked in the genome also contributes to the enrichment signature observed. The close clustering of *IL23R* and *IL12RB2* (chr1p31) is consistent with this. This concordance of expression for closely linked genes also suggests, in some cases, a limitation in defining a single gene driving what are often complex, extended and multiple disease associations within a particular locus.

### Association, Expression and Functional Overlap between IL-23 and IL-12 Pathways

Within the *IL23R*-*IL12RB2* locus, several independent SNPs have been associated with multiple immune-mediated diseases. A protective missense mutation in *IL23R*, Arg381Gln, has been reported to be a loss-of-function allele [25], [26], indicating that increased IL-23 signaling increases immune-mediated disease risk. Multiple independent association signals are observed within the *IL23R* gene region and the intergenic region between *IL23R* and *IL12RB2*. While cis-acting eQTLs are present within *IL12RB2* [27], these eSNPs are not correlated with disease association signals. No eQTLs and allelic imbalance have been reported for *IL23R*. The absence of correlations between mRNA levels and single SNPs associated with disease could reflect more complex mappings between disease-associated polymorphisms and gene expression. One possibility is that haplotypes in the *IL23R*-*IL12RB2* gene region confer greater disease risk; consistent with this, GWAS-derived haplotypes provide superior predictive capacity for disease risk in Crohn’s disease compared to single-marker models [28] suggesting improved biologic modeling with haplotypes. Additional possibilities are that disease-associated polymorphisms may modulate gene expression only in specific biologic contexts (e.g. cellular activation), that the key alteration in gene expression may be post-transcriptional, or that disease-associated polymorphisms may modulate relative expression of alternative isoforms.

The abundance of an *IL23R* isoform terminating at exon 6, which would not encode the transmembrane domain, suggests that *IL23R* isoforms play a significant role in gene regulation. The abundance of sequence reads in consensus intron 6 indicates that transcripts terminating in an extended exon 6 are highly prevalent. A recent study reported an isoform lacking the exon 9, transmembrane domain (Δ9) [29]. This alternative isoform would encode for a soluble form of *IL23R*, and comprised approximately 10% of *IL23R* isoforms. However, this study performed mass spectrometry on amplified cDNA using primers from exons 2 and 11, and could therefore not identify the major isoform terminating in an extended exon 6. Whether the non-coding disease-associated polymorphisms in the *IL23R* gene region modulate the relative fraction of exon 6 terminating compared to full-length *IL23R* will be the subject of future studies; ongoing, large scale, fine-mapping association studies performed using Immunochip may provide important insight.

IL-12 and IL-23 play central, proliferative roles in Th1 (IFNγ-expressing) and Th17 (IL-17 expressing) cells, respectively. Expression patterns of *IL23R* and *IL12RB2* are similar across CD4+ T cell subsets, with both induced in Th17-enriched and Th1 subsets, relative to Th17-negative and CD4+ naïve cells, respectively. In fact, expression of *IL23R* is relatively higher in vitro differentiated Th1 compared to Th17-enriched subsets. In CD4+CD45Rb^HI^ *IL23R−/−* T cell transfer models, *IL23R−/−* T cells demonstrated normal Th1 differentiation. However, in the intestine CD4+CD45Rb^HI^ *IL23R−/−* T cells showed impaired proliferation, decreased accumulation, and reduced expression of IL-17+IFNγ+ cells [19]. Therefore, multiple factors regulate the downstream consequences to T cell subset outcomes in addition to the regulated expression of select proteins.

Importantly, Th17 cells demonstrate substantial plasticity after their initial commitment to the Th17 program [30], [31]; IL-12 can extinguish RORγt and IL17 expression in Th17 cells [15] through covalent histone modifications resulting in epigenetic silencing of the *IL17A*-*IL17F* and *RORC* genes and enhancement of *IFN*γ expression. In fact, using cell fate mapping in the experimental autoimmune encephalomyelitis (EAE) model, proinflammatory cytokines in the spinal cord were produced almost exclusively by “ex-Th17 cells” [32]. The specialized, sequential functions of the Th17 and Th1 pathways [33] in jointly mediating mucosal inflammation have yet to be fully defined. It is possible that the Th17 pathway plays an essential initiating and amplifying role in mucosal inflammation through its joint expression of the chemokine receptor, CCR6 [34], and its ligand, CCL20, which results in efficient transport of effector Th17 cells to mucosal sites. Consistent with this, demethylation of IL17A and CCL20 promoter CpG sites plays a key role in initial Th17 program commitment (Figure 4C–D). The presence of repressive H3K27me3 epigenetic marks throughout the CCR6 gene region in murine-derived Th1 cells [35] further supports the concept that a key feature of Th17 cells involves its efficient transport to mucosal sites. Teleologically, it has been proposed that the versatility of Th17 cells to adjust its effector program as needed allow them to respond most efficiently to early and late immune clearance requirements, as well as to a broad range of mucosal pathogens which ultimately might confer significant evolutionary benefit [30].

### Toward a Comprehensive Molecular Integration of Disease Association Signals and Differential Gene Expression

The enrichment of Th17-Th1 expression signatures for genes contained within immune-mediated genetic loci implies, but does not establish, a directionality of disease-associated polymorphisms on gene expression and function. A key next step in this regard is the performance of fine-mapping studies using large case-control cohorts to refine independent and dependent SNPs and haplotypes that account for observed association signals. RNASeq data provides a useful adjunct to eQTL mapping [36] by providing molecular mapping to variable gene expression. Alternative isoforms, post-transcriptional regulation and regulation in select biologic conditions likely also contribute to disease-associated regulation of gene expression. Integration of allele and haplotype-based variable gene expression with disease association results from large fine-mapping studies will provide an important foundation toward developing increasingly refined models of gene expression and disease networks.

## Materials and Methods

### Cell Preparation

This study has been approved by Yale University Institutional Review Boards. A written informed consent has been obtained from each individual healthy donor, and the consent procedure has also been approved by Yale University Institutional Boards. Peripheral blood mononuclear cells were isolated from 5 individual healthy donors with Ficoll separation. CD4+ T cells were enriched by negative selection (Miltenyi Biotech) and CD4+CD25-CD45RO+CCR6+CD161+, CD4+CD25-CD45RO+CCR6-CD161- and CD4+CD25-CD45RO-CD62L+ naïve T cells were sorted by FACS Aria (BD Bioscience). For Th1 differentiation, naive T cells were activated with plate-bound anti-CD3 mAb (10µg/ml) and soluble anti-CD28 mAb (1.0 µg/ml) in serum-free media supplemented with recombinant IL-12 (5 ng/ml) and anti-IL-4 (10 µg/ml) for 7 days. Sorted CD4^+^ memory T cell subsets were activated with plate-bound anti-CD3 mAb and soluble anti-CD28 mAb, and supplemented with IL-23 (20 ng/ml) and IL-1β (10 ng/ml) for 7 days. Intracellular staining was performed by using the Cytofix/Cytoperm buffer set (BD Biosciences) according to the manufacturer’s instructions. Briefly, cells were incubated for 5 hours with PMA (50 ng/ml), ionomycin (500 ng/ml) and GolgiPlug (BD Biosciences, CA), then permeabilized with Cytofix/Cytoperm buffer and stained with FITC-conjugated anti-IFNγ (BD Biosciences) and APC-conjugated anti-IL-17 (eBioscience, CA).

### Total RNA Isolation, Microarray, Definition of Immune-mediated Gene Loci, Enrichment and Cluster Analysis

Total RNA was isolated from naïve CD4+ T cells, Th1, Th17-enriched and Th17-negative memory CD4+ T cells using the RNeasy Mini Kit (Qiagen) with DNase treatment. Naïve and Th1 cells were from 4 individuals. Th17-enriched and Th17-negative memory CD4+ T cells were from 5 individuals, and 4 of them are the same individuals as the Naïve and Th1 group. High quality (RIN >8.5) RNA was hybridized to the Affymetrix GeneChipR Human Gene 1.0 ST expression arrays for gene expression profiling. RMA normalization [37] of the intensity data was performed using the Partek Genomics Suite. All microarray data is MIAME compliant and the raw data has been deposited in GEO database. The GEO accession number is GSE 32901.

Immune-mediated disease loci were defined by the inclusion as a fine-mapping disease locus on the custom-designed Immunochip. Loci were included if genome-wide significant evidence for disease association was observed in at least one immune-mediated disease (Crohn^1^s disease, ulcerative colitis, celiac disease, psoriasis, type 1 diabetes mellitus, rheumatoid arthritis, multiple sclerosis, ankylosing spondylitis, systemic lupus erythematosus). All European ancestry 1000 Genomes SNPs within 0.1 cM of the peak association signals were included in the fine-mapping loci, and 1387 transcripts within the fine-mapping boundaries were included in the enrichment analyses. For each possible ordered pair of 2 cell types, (cell type 1, cell type 2), where cell type belongs to the set (naive, in vitro differentiated Th1, Th17-enriched and Th17-negative), we measure the degree of enrichment between a set of 1387 transcripts, chosen for to their proximity to immune-mediated disease associated loci, and the set of up-regulated transcripts in cell type 1 relative to cell type 2. Fisher’s exact test was used to estimate enrichment P-values using the following steps:

- Calculate the P-values for upregulation of cell type 1 relative to cell type 2 for 18,524 transcripts (that are common to both RNASeq and microarray).
- From this set of 18,524 transcripts, find a subset of transcripts, S, which are not related to autoimmune disease loci, with the property that the median gene expression of cell type 1 and cell type 2 for transcripts within this set, S, equals the median gene expression within the 1387 immune mediated disease transcripts. Next merge this set with the 1387 immune mediated disease transcripts to create a ‘background’ set of transcripts appropriate for the enrichment test.
- Construct a 2 x 2 table of counts which assigns each of the background transcripts to one of 4 cells depending on whether it is among the 5% most differentially expressed transcripts (according to P-value), and whether the transcript is within the 1387 autoimmune transcripts or not.
- Calculate the Fisher’s exact test P-value associated with the table constructed in (iii).

### Transcriptome Sequencing

RNA libraries were prepared according to the manufacturer’s recommended protocol (Illumina, CA). Total RNA samples of Naïve, in vitro differentiatedTh1, Th17-enriched and Th17-negative CD4+ T cells from 4 individual healthy controls were transcribed to cDNA. cDNA samples were then sheared by nebulization (35 psi, 6 min). Duplexes were blunt ended (large Klenow fragment, T4 polynucleotide kinase and T4 polymerase) and a single 3′adenosine moiety was added using Klenow exo^−^ and dATP. Illumina adapters, containing primer sites for flow cell surface annealing, amplification and sequencing, were ligated onto the repaired ends of the cDNA. Gel electrophoresis was used to select for DNA constructs 200–250 base pairs in size, which were subsequently amplified by 18 cycles of PCR with Phusion polymerase. These libraries were denatured with sodium hydroxide and diluted to 3.5 pM in hybridization buffer for loading onto a single lane of an Illumina GA flow cell. Cluster formation, primer hybridization and sequencing reactions were according to the manufacturer’s protocol. High throughput sequencing was performed using paired end, 75 base pair reads. Two flow lanes were used for each cDNA sample, yielding an average of 103.3 million reads per sample.

### RNASeq Mapping, Estimates of Differential Expression and Isoform Abundance

Tophat v1.3.3 [38] was used to align the RNAseq reads to the hg19 genome. RNASeq gene expression was measured for each gene from version 59 of the Ensembl database by Mapped Fragments per Kilobase of Exon model per Million mapped reads (FPKM) calculated via Cufflinks v1.2.1. The Affymetrix annotation for the Human HuGene 1.0 array was downloaded and used to annotate the microarray probes. Subsequently, microarray intensity was calculated as the RMA-normalized log intensity for a single chosen probe in a given gene region. Microarray intensity and FPKM were matched on common gene name for 18,524 genes, and plotted against each other (Figure S1).

We used the program Cuffdiff to test for differential transcript expression in each pair of cell lines. When samples were paired, it was necessary to analyze each sample individually in Cufflinks before combining the resulting P-values using the Fisher method to get a single P-value for differential expression for each gene. We made an exception to this rule for genes where the estimated log fold changes for the 4 samples did not all have the same sign. These genes were assigned a P-value of 1. P-values for differential expression from the microarray data were calculated via paired t-tests. The QQ plot (Figure 2) was created by plotting the observed ordered negative log_10_ P-values for the RNASeq (or microarray) data against the ordered negative log_10_ P-values that would be expected if no genes were differentially expressed.

### Quantitative Methylation Studies

Quantitative DNA methylation analysis was performed at the Keck Core Facility at Yale University using the MassARRAY EpiTYPER system (Sequenom, CA) according to the manufacturer’s instructions. Genomic DNA samples were bisulfite treated to convert non-methylated cytosine into uracil using the EZ DNA methylation kit (Zymo Research, CA), followed by PCR amplification using T7-promotor tagged reverse primers. After shrimp alkaline phosphatase treatment, in vitro transcription was performed, and the generated transcript was subjected to an enzymatic base specific cleavage. The resulting fragments differ in size and mass depending on the sequence changes generated through bisulfite treatment. The fragment masses were determined by MALDI-TOF MS and the EpiTYPER software used to estimate percentages of methylation at CpG sites for each analyzed fragment.

### 3′ Rapid Amplification of cDNA Ends (3′ RACE)

3′ Race was performed with FirstChoice RLM-RACE Kit (Ambio by Applied Biosystem, CA) according to manufacturer’s protocol. 3′ Race outer and inner primers were designed from Exon 6 with Primer3 Plus, the first and nested PCR were done using an annealing temperature of 60°C. The highly expressed fragments after nested PCR were purified with QIAquick Gel Extraction Kit (Qiagen, CA), and then Sanger sequenced by using ABI 3730XL DNA Analyzer and sequence scanner v1.0 from ABI.

## Supporting Information

Figure S1 **RNASeq gene expression, measure by log2 FPKM, is highly** **correlated with microarray gene expression measured by log2 normalized intensity.** (Pearson correlation ranging from 0.83 to 0.85 in naïve, in vitro differentiated Th1, Th17-negative, and Th17-enriched CD4+ T cell subsets.
(TIFF)

Figure S2 **A. Hierarchical clustering, based on normalized microarray intensity, was performed for a set of 147 transcripts.** The transcripts were selected to show at least a 1.5 fold up-regulation, and a P-value for differential expression less than 0.05 in the Th17-enriched cell subset compared to the Th17-negative cell subset. A total of 25 transcripts, associated with various autoimmune diseases, are designated with asterisks (*) on the right hand side of the heatmap. B. Disease-associated transcripts demonstrating increased expression in both Th17-enriched and Th1 cells: IL1R2, interleukin 1 receptor 2; PFKFB3, 6-phophofructo-2-kinase/fructose-2,6-biphosphatase 3; IL18RAP, interleukin 18 receptor accessory protein; IL18R1, interleukin 18 receptor 1; IL12RB2, interleukin 12 receptor, beta2; IL23R, interleukin 23 receptor; ITGAX, integrin, alpha X (complement 3 receptor 4 subunit); VDR, vitamin D (1, 25-dihydroxyvitamin D3) receptor; SMOX, spermine oxidase; ATP6V0A1, ATPase, H+ transporting, lysosomal V0 subunit. C. Disease-associated transcripts demonstrating increased expression solely in Th17-enriched cells: KLRB1, killer cell lectin-like receptor subfamily B, member 1; IL17F, interleukin 17, F isoform; CCR6, chemokine (C-C motif) receptor 6; CAMTA1, calmodulin binding transcription activator 1; IL1R1, interleukin 1 receptor 1.
(TIFF)

Figure S3 **A. Vista genome browser view showing conservation scores near the** ***IL23R*** **promoter.** In the *IL23R* promoter sequences, italicized red CpGs were included in the methylation analysis and SNPs are shown in blue. B. Fractions of methylation at conserved CpG promoter sites estimated by mass spectrometry (N = 5) for *IL22*, *RORC*, *IL7R*, *IL18RAP and IL17F* promoters. A paired t-test were used to test for differential methylation for the comparisons: naïve vs. Th1 and Th17-negative vs. Th17-enriched; *P<0.05, **P<0.01, ***P<0.001.
(TIFF)

Figure S4 **Confirmation of sequence contiguity for** ***IL23R*** **isoforms terminating in the extended exon 6 region.** The reverse primer (5′ GGGTTGAAAAGCAAATTATTGGTAACTA - 3′) was designed from the extended exon 6 region, with forward primers designed from exons 1 through 6. The forward primers were: Exon 1, 5′ - GGTCAAGCGATCACTGAACTTAGA -3′; Exon 2, 5′ - CCTTTACATACTCTTCAGCTGGTGTC -3′; Exon 3, 5′ - TATTGCCAAGCAGCAATTAAGAAC -3′; Exon 4, 5′ - AGAAGAGCAACATGATCTCACCTCAA -3′; Exon 5, 5′ - CAAGGCTACAACAAACCAAACTT -3′; Exon 6, 5′ - CAAGGCTACAACAAACCAAACTT - 3″.
(TIFF)

Table S1 Known GWAS disease associations within upregulated transcripts in Th17-enriched cells compared to Th17-negative CD4+ T cells according to RNASeq.
(XLS)

Table S2 Known GWAS disease associations within upregulated transcripts in Th17-enriched cells compared to Th17-negative CD4+ T cells according to Microarray.
(XLS)
