# Supplementary figures and images for: Effector CD4+ T Cell Expression Signatures and Immune-Mediated Disease Associated Genes
Source: PLoS One. 2012 Jun 8;7(6):e38510. doi: 10.1371/journal.pone.0038510 (PMC3371029; doi:10.1371/journal.pone.0038510)

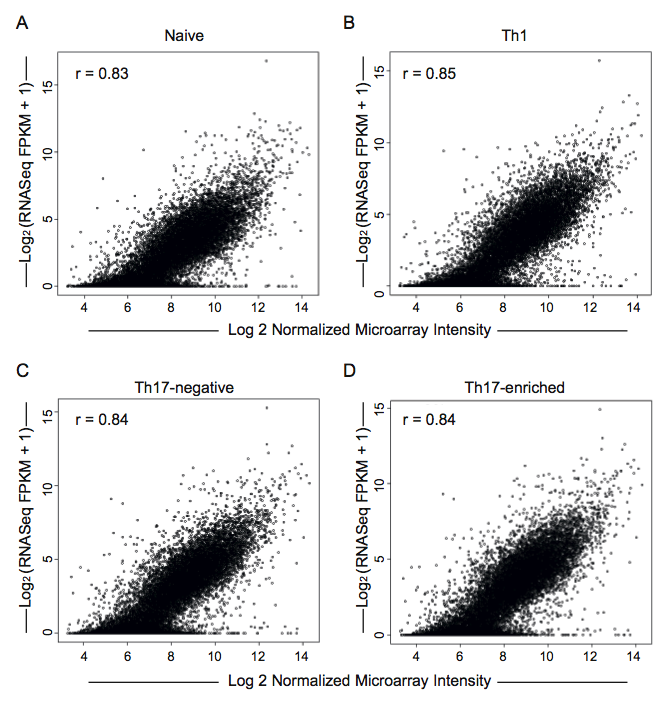

Supplement: Figure S1 — RNASeq gene expression, measure by log2 FPKM, is highly correlated with microarray gene expression measured by log2 normalized intensity. (Pearson correlation ranging from 0.83 to 0.85 in naïve, in vitro differentiated Th1, Th17-negative, and Th17-enriched CD4+ T cell subsets. (TIFF) [file pone.0038510.s001.tiff]

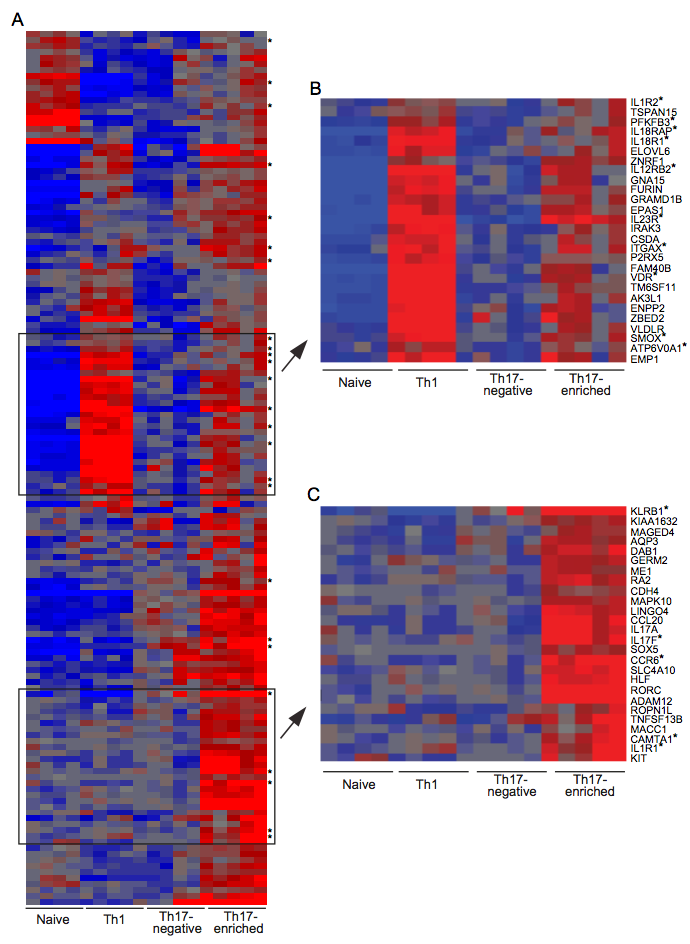

Supplement: Figure S2 — A. Hierarchical clustering, based on normalized microarray intensity, was performed for a set of 147 transcripts. The transcripts were selected to show at least a 1.5 fold up-regulation, and a P-value for differential expression less than 0.05 in the Th17-enriched cell subset compared to the Th17-negative cell subset. A total of 25 transcripts, associated with various autoimmune diseases, are designated with asterisks (*) on the right hand side of the heatmap. B. Disease-associated transcripts demonstrating increased expression in both Th17-enriched and Th1 cells: IL1R2, interleukin 1 receptor 2; PFKFB3, 6-phophofructo-2-kinase/fructose-2,6-biphosphatase 3; IL18RAP, interleukin 18 receptor accessory protein; IL18R1, interleukin 18 receptor 1; IL12RB2, interleukin 12 receptor, beta2; IL23R, interleukin 23 receptor; ITGAX, integrin, alpha X (complement 3 receptor 4 subunit); VDR, vitamin D (1, 25-dihydroxyvitamin D3) receptor; SMOX, spermine oxidase; ATP6V0A1, ATPase, H+ transporting, lysosomal V0 subunit. C. Disease-associated transcripts demonstrating increased expression solely in Th17-enriched cells: KLRB1, killer cell lectin-like receptor subfamily B, member 1; IL17F, interleukin 17, F isoform; CCR6, chemokine (C-C motif) receptor 6; CAMTA1, calmodulin binding transcription activator 1; IL1R1, interleukin 1 receptor 1. (TIFF) [file pone.0038510.s002.tiff]

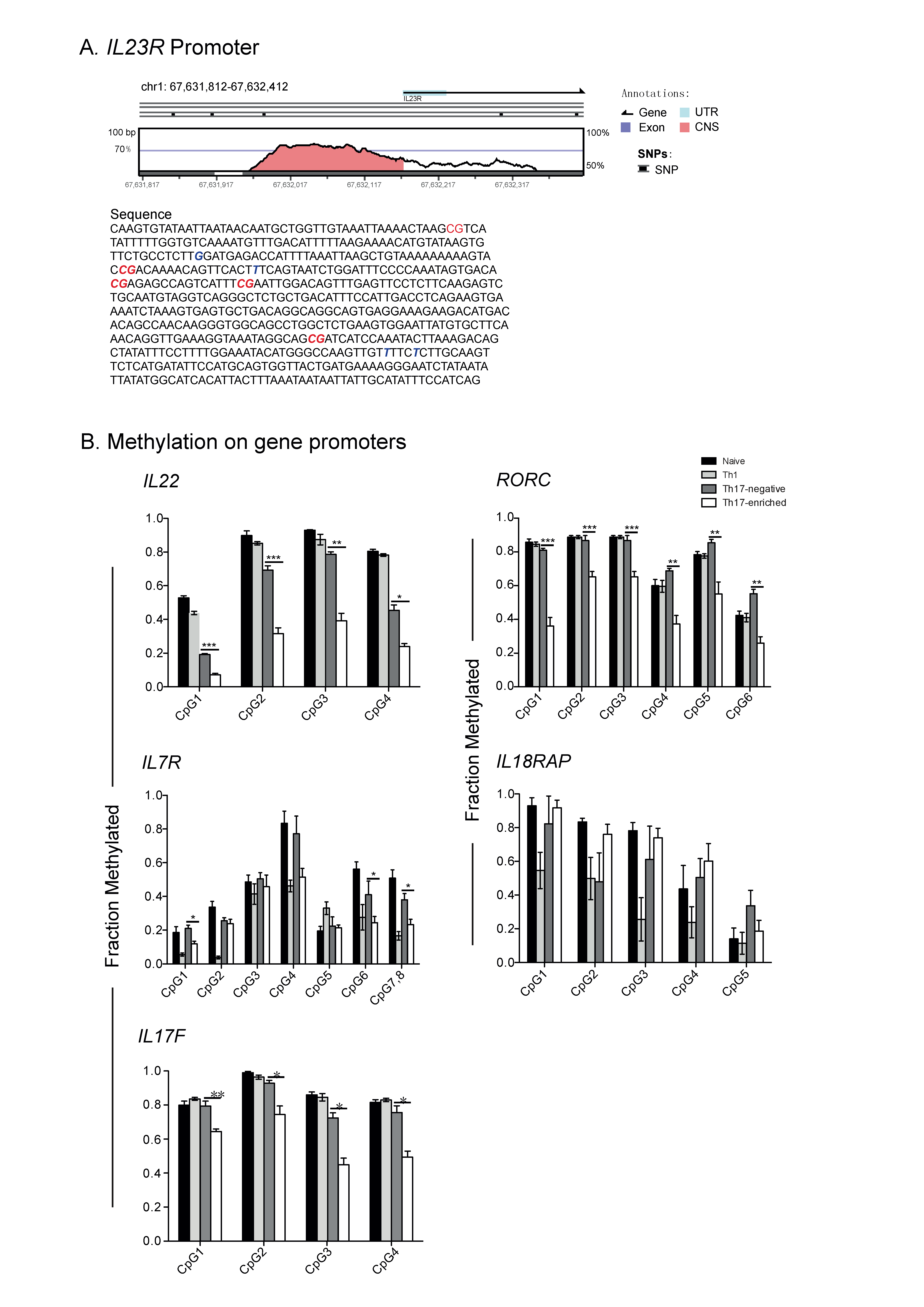

Supplement: Figure S3 — A. Vista genome browser view showing conservation scores near the IL23R promoter. In the IL23R promoter sequences, italicized red CpGs were included in the methylation analysis and SNPs are shown in blue. B. Fractions of methylation at conserved CpG promoter sites estimated by mass spectrometry (N = 5) for IL22, RORC, IL7R, IL18RAP and IL17F promoters. A paired t-test were used to test for differential methylation for the comparisons: naïve vs. Th1 and Th17-negative vs. Th17-enriched; *P<0.05, **P<0.01, ***P<0.001. (TIFF) [file pone.0038510.s003.tiff]

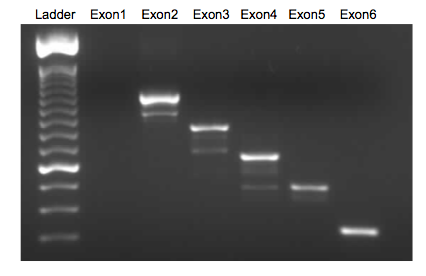

Supplement: Figure S4 — Confirmation of sequence contiguity for IL23R isoforms terminating in the extended exon 6 region. The reverse primer (5′ GGGTTGAAAAGCAAATTATTGGTAACTA - 3′) was designed from the extended exon 6 region, with forward primers designed from exons 1 through 6. The forward primers were: Exon 1, 5′ - GGTCAAGCGATCACTGAACTTAGA -3′; Exon 2, 5′ - CCTTTACATACTCTTCAGCTGGTGTC -3′; Exon 3, 5′ - TATTGCCAAGCAGCAATTAAGAAC -3′; Exon 4, 5′ - AGAAGAGCAACATGATCTCACCTCAA -3′; Exon 5, 5′ - CAAGGCTACAACAAACCAAACTT -3′; Exon 6, 5′ - CAAGGCTACAACAAACCAAACTT - 3″. (TIFF) [file pone.0038510.s004.tiff]
